# Surgeon- and procedure-specific variability in operative-time learning curves during parallel implementation of robotic colorectal surgery

**DOI:** 10.3389/fonc.2026.1905964

**Published:** 2026-09-11

**Authors:** Zsolt Madarasz, Kira Baginski, Annika Hoyer, Julia Michel, Krzysztof Nowakowski, Michael Leitz, Fabian Nimczewski, Jens Hoeppner

**Affiliations:** 1 Department of Surgery, Bielefeld University, Medical School and University Medical Center OWL, Detmold, Germany; 2 Biostatistics and Medical Biometry, Medical School OWL, Bielefeld University, Bielefeld, Germany

**Keywords:** CUSUM analysis, learning curve, robotic colorectal surgery, robotic colorectal surgery training, surgeon variability

## Abstract

**Background:**

Robotic colorectal surgery is associated with learning trajectories that may vary according to procedural complexity and individual surgeon experience. Although robotic colorectal learning curves have been extensively investigated in single-surgeon series, limited data exist regarding inter-surgeon variability during the implementation of multiple robotic colorectal procedures within the same institution. This study evaluated surgeon- and procedure-specific learning curves during the progressive institutional implementation of a robotic colorectal surgery program.

**Methods:**

We reviewed 369 consecutive patients who underwent elective robotic colorectal resections between 2018 and 2025. Operative performance of three primary console surgeons was analyzed for robotic right colectomy (RRC) with complete mesocolic excision (CME), robotic anterior resection (RAR), and robotic low anterior resection (RLAR). Learning curves were assessed using cumulative sum (CUSUM) analysis of skin-to-skin operative times. Risk-adjusted CUSUM (RA-CUSUM) analysis was used to account for selected patient-related factors potentially influencing operative time.

**Results:**

CUSUM analysis demonstrated substantial surgeon- and procedure-specific variability in operative learning trajectories. RRC generally demonstrated earlier operative-time stabilization, with CUSUM turning points ranging from 8 to 18 procedures. RLAR demonstrated the greatest variability in operative learning trajectories and the highest operative times, with CUSUM turning points ranging from 16 to 27 procedures, whereas RAR showed intermediate learning trajectories. Despite heterogeneous CUSUM profiles between surgeons and procedures, overall perioperative and oncological outcomes were acceptable across the study cohort.

**Conclusion:**

Operative learning trajectories in robotic colorectal surgery demonstrate considerable variability according to both surgeon and procedure, even within a standardized institutional robotic program. While RRC generally showed earlier operative-time stabilization, RLAR was characterized by higher operative times and greater variability in operative learning trajectories. These findings suggest that operative learning trajectories vary substantially between surgeons and procedures and should not be interpreted using fixed case-number thresholds alone.

## Introduction

Robotic-assisted surgery has become an integral component of modern minimally invasive colorectal cancer treatment. The platform’s technical advantages, including stable three-dimensional visualization, articulated instruments, and improved ergonomics, are particularly relevant during complex pelvic dissection and intracorporeal reconstruction. Consequently, robotic surgery is increasingly applied to technically demanding colorectal procedures, including robotic anterior resection (RAR), robotic low anterior resection (RLAR), and robotic right colectomy (RRC) with complete mesocolic excision (CME).

Despite these advantages, implementation of robotic colorectal surgery is associated with an operative learning process influenced by procedural complexity, surgeon experience, and training structures (1, 2). Previous studies evaluating operative performance using cumulative sum (CUSUM) methodology have predominantly focused on single procedures or single-surgeon experiences and have demonstrated considerable variability in operative learning trajectories (3–5).

Our previous studies examined the initial institutional implementation of robotic colorectal surgery within a structured training environment. Using CUSUM analysis, we identified procedure-specific differences in operative learning trajectories for RAR, RLAR, and RRC in a single-surgeon series (6). In a subsequent analysis, RRC with CME, central vascular ligation, and intracorporeal anastomosis was associated with favorable perioperative and oncological outcomes during early institutional implementation (7). However, these studies primarily reflected single-surgeon or procedure-specific experiences and did not address whether operative learning trajectories are reproducible across different surgeons performing multiple procedures within the same institutional program.

The present study therefore expands these previous observations by evaluating surgeon- and procedure-specific operative learning trajectories across three robotic colorectal procedures within a multi-surgeon institutional program. Using CUSUM and risk-adjusted CUSUM (RA-CUSUM) analyses of operative time, we aimed to assess inter-surgeon variability, procedure-specific learning characteristics, and changes in operative performance during institutional implementation.

## Materials and methods

### Study design and patient population

This retrospective single-center study analyzed a prospectively maintained database of consecutive patients undergoing elective robotic colorectal surgery between June 2018 and December 2025 at a tertiary referral colorectal center. A total of 369 patients were included in the analysis.

Inclusion criteria comprised consecutive adult patients undergoing RRC with CME, RAR, or RLAR performed by one of the three primary console surgeons during institutional implementation of robotic colorectal surgery. Malignant indications included right-sided colon cancer, left-sided colon cancer, and rectal cancer across all tumor stages. Patients undergoing RAR for diverticular disease were also included.

Exclusion criteria comprised emergency procedures, non-robotic operations, and procedures not corresponding to the predefined study procedures (RRC, RAR, RLAR).

The primary objective was to evaluate surgeon- and procedure-specific operative learning trajectories during progressive institutional adoption of robotic colorectal surgery. Secondary objectives were to compare operative learning trajectories between surgeons and procedures and to descriptively assess perioperative and oncological outcomes across the study cohort.

### Robotic training program and surgical implementation

Robotic colorectal surgery was introduced at our institution using the da Vinci X platform in June 2018. The da Vinci Xi and da Vinci 5 systems were introduced in 2025, toward the end of the study period; consequently, the majority of procedures were performed using the da Vinci X system. Case-level information on the use of the Xi and da Vinci 5 systems was not systematically recorded and could therefore not be reliably assigned to individual surgeons or procedures. Operative technique, port placement, docking strategy, and procedural principles remained standardized across platforms.

All three surgeons had several years of experience in advanced laparoscopic colorectal surgery before initiating robotic colorectal surgery. Before independent console surgery, each surgeon completed a structured institutional training pathway comprising simulator-based console training, dry- and wet-lab exercises, bedside assistance, and supervised robotic colorectal procedures according to the Intuitive Surgical training curriculum. Initial console procedures were performed under direct proctoring.

During the initial implementation phase, selected procedures were performed using the dual-console platform with two colorectal surgeons present. The primary console surgeon was defined as the surgeon responsible for the procedure and performing the majority of the operation, including the principal resection steps. The second surgeon primarily observed the procedure for training purposes and only occasionally performed selected operative steps. For all surgeon-specific learning curve analyses, each procedure was attributed to the predefined primary console surgeon.

Implementation did not occur simultaneously among the three surgeons. Surgeon 1 initiated RLAR in 2018, whereas Surgeon 3 initiated RLAR and RAR in 2021 and Surgeon 2 in 2022. RRC was introduced later and within a narrower time frame, beginning in 2021 for Surgeon 1 and in 2022 for Surgeons 2 and 3.

### Surgical procedures

RRC was performed for right-sided colonic lesions extending to the proximal transverse colon. The procedure included CME with central vascular ligation of the ileocolic vessels and intracorporeal ileocolic anastomosis.

RAR was defined as resection of the upper rectum with partial mesorectal excision (PME) and colorectal anastomosis. RLAR was defined as resection of mid- and low-rectal lesions with total mesorectal excision (TME) and low colorectal or coloanal anastomosis. Both procedures included high ligation of the inferior mesenteric artery and vein according to standardized oncological principles.

Patients undergoing RAR for diverticular disease were also included, as these procedures followed the same standardized robotic operative approach and dissection planes as oncological anterior resections.

Throughout the study period, the operative technique for each procedure remained standardized, including port placement, docking strategy, dissection planes, and anastomotic technique. Diverting stoma formation was performed according to clinical indications and, when performed, was included in the skin-to-skin operative time.

### Data collection and outcome measures

Demographic, perioperative, postoperative, and histopathological variables were extracted from the prospectively maintained institutional database. Baseline variables included age, sex, body mass index (BMI), American Society of Anesthesiologists (ASA) classification, indication for surgery, previous abdominal surgery, smoking status, anticoagulant treatment, and relevant comorbidities.

Perioperative outcomes included skin-to-skin operative time, conversion to open surgery, intraoperative complications, postoperative complications according to the Clavien–Dindo classification (8), anastomotic leakage, 30-day reoperation, 30-day mortality, and postoperative length of hospital stay (days).

Histopathological outcomes included pathological TNM stage, UICC stage, resection status, lymph node yield, CME specimen quality in RRC procedures (9), TME specimen quality in RLAR procedures (10), and circumferential resection margin (CRM) status in RLAR procedures.

### Statistical analysis

The patient characteristics, perioperative, postoperative, and histopathological outcomes were initially analyzed descriptively. Continuous variables are presented in terms of mean (standard deviation) or median (Q1-Q3), whereas categorical variables are reported as absolute numbers and percentages.

To assess and compare changes in surgical performance over time by procedure, the CUSUM method was used, with skin-to-skin operative time serving as the primary performance metric. The target value was defined as the mean operative time per procedure, calculated separately for each surgeon. For each case, the difference between the observed operative time and this reference value was determined, and these differences were cumulatively summed to generate individual CUSUM learning curves for each surgeon and procedure.

The CUSUM score *C_i_* for the i-th consecutive surgery was calculated using the following formula (11):


$$C_{i}=\sum_{j=1}^{i}\left(X_{j}-\bar{X}\right)$$

Where *X_j_* denotes the operative time of the j-th surgery and $\bar{X}$ represents the mean operative time across all surgeries for the respective surgeon–procedure combination.

Turning points were identified by visual inspection as the highest point of each CUSUM curve, corresponding to the transition from an increasing to a decreasing cumulative operative-time trend. Turning points were considered descriptive indicators of changes in operative-time trends and were not interpreted as validated thresholds of technical or overall surgical proficiency.

Because operative time may be influenced by patient-related factors, a RA-CUSUM analysis was also performed. Rather than assuming a fixed mean operative time for each surgeon–procedure combination, a separate multivariable linear regression model was developed for each combination to estimate the expected operative time for each individual surgery. Accordingly, the fixed mean operative time $\bar{X}$ in the CUSUM formula was replaced by $X_{j}^{exp}$ representing the model-based expected operative time for the j-th surgery. The models incorporated relevant predictors, including sex, age, BMI, ASA score, prior abdominal surgery, and smoking status. Descriptive characteristics of the covariates included in the regression models are provided in **Supplementary Table 1**, while corresponding regression diagnostic plots are presented in **Supplementary Figures 2**–**10**. The differences between observed and expected operative times were then cumulatively summed to construct individualized RA-CUSUM learning curves for each surgeon and procedure. A complete-case approach was used, as no data were missing in either the descriptive or CUSUM analyses. Therefore, no imputation of missing data was required. All statistical analyses were performed using R version 4.6.1.

## Results

### Patient characteristics

A total of 369 consecutive patients undergoing elective robotic colorectal surgery between 2018 and 2025 were included in the analysis. Procedures were performed by three surgeons, with 155 operations performed by Surgeon 1, 93 by Surgeon 2, and 121 by Surgeon 3. The overall median age was 71 years (Q1-Q3: 61–79), and 58.0% of patients were male. Median BMI was 26 kg/m² (Q1-Q3: 24–29), and 90.7% of patients were classified as ASA II or III.

Rectal cancer was the most common indication (42.5%), followed by right-sided colon cancer (25.7%), left-sided colon cancer (17.9%), and sigmoid diverticulitis (13.8%). Previous abdominal surgery was present in 26.8% of the overall cohort. Detailed baseline characteristics, procedural distribution, and comorbidity profiles by surgeon are presented in **Table 1**.

**Table 1:** Baseline characteristics.

Variable	Surgeon 1 (n=155)	Surgeon 2 (n=93)	Surgeon 3 (n=121)	Overall (n=369)
Age (in years)				
Mean (SD)	70.0 (11.5)	69.9 (12.8)	68.8 (11.6)	69.6 (11.8)
Median [Q1, Q3]	72.0 [61.5, 79.0]	71.0 [61.0, 81.0]	70.0 [62.0, 78.0]	71.0 [61.0, 79.0]
Sex				
Female	62 (40.0%)	46 (49.5%)	47 (38.8%)	155 (42.0%)
Male	93 (60.0%)	47 (50.5%)	74 (61.2%)	214 (58.0%)
BMI kg/m²				
Mean (SD)	26.8 (4.54)	27.5 (5.85)	26.3 (4.68)	26.8 (4.96)
Median [Q1, Q3]	26.0 [24.0, 29.0]	27.0 [24.0, 30.0]	26.0 [23.0, 28.0]	26.0 [24.0, 29.0]
ASA Score				
I	5 (3.2%)	3 (3.2%)	9 (7.4%)	17 (4.6%)
II	70 (45.2%)	36 (38.7%)	51 (42.1%)	157 (42.5%)
III	74 (47.7%)	49 (52.7%)	55 (45.5%)	178 (48.2%)
IV	6 (3.9%)	5 (5.4%)	6 (5.0%)	17 (4.6%)
Indication				
Left colon cancer	27 (17.4%)	11 (11.8%)	28 (23.1%)	66 (17.9%)
Rectal cancer	87 (56.1%)	25 (26.9%)	45 (37.2%)	157 (42.5%)
Right colon cancer	35 (22.6%)	32 (34.4%)	28 (23.1%)	95 (25.7%)
Sigmoid diverticulitis	6 (3.9%)	25 (26.9%)	20 (16.5%)	51 (13.8%)
**Previous abdominal surgery**	42 (27.1%)	31 (33.3%)	26 (21.5%)	99 (26.8%)
Comorbidities				
Type 2 diabetes mellitus	35 (22.6%)	12 (12.9%)	14 (11.6%)	61 (16.5%)
Coronary artery disease	18 (11.6%)	11 (11.8%)	16 (13.2%)	45 (12.2%)
Cardiac insufficiency	33 (21.3%)	19 (20.4%)	22 (18.2%)	74 (20.1%)
Arterial hypertension	92 (59.4%)	59 (63.4%)	67 (55.4%)	218 (59.1%)
Renal insufficiency	5 (3.2%)	3 (3.2%)	8 (6.6%)	16 (4.3%)
Chronic obstructive pulmonary disease (COPD)	8 (5.2%)	8 (8.6%)	6 (5.0%)	22 (6.0%)
Anticoagulant treatment	46 (29.7%)	28 (30.1%)	34 (28.1%)	108 (29.3%)
Smoking	25 (16.1%)	21 (22.6%)	27 (22.3%)	73 (19.8%)

### Perioperative outcomes

Surgeon 1 performed predominantly RLAR procedures (56.1%), whereas Surgeon 2 had a higher proportion of RAR procedures and diverticulitis cases. Overall conversion to open surgery occurred in 1.9% of cases, and intraoperative complications occurred in 0.5%. Major postoperative complications (Clavien–Dindo ≥ III) occurred in 8.7% of patients, while anastomotic leakage was observed in 3.5%. The 30-day reoperation rate and 30-day mortality risk were 8.4% and 1.4%, respectively.

Median length of hospital stay was 8 days (Q1-Q3: 6–11). Perioperative outcomes stratified by surgeon are presented descriptively in **Table 2**; no formal between-surgeon comparisons were performed.

**Table 2:** Perioperative outcomes.

Variable	Surgeon 1 (n=155)	Surgeon 2 (n=93)	Surgeon 3 (n=121)	Overall (n=369)
Procedure				
Robotic right colectomy (RRC)	35 (22.6%)	32 (34.4%)	28 (23.1%)	95 (25.7%)
Robotic anterior resection (RAR)	33 (21.3%)	36 (38.7%)	48 (39.7%)	117 (31.7%)
Robotic low anterior resection (RLAR)	87 (56.1%)	25 (26.9%)	45 (37.2%)	157 (42.5%)
Conversion to open surgery	3 (1.9%)	2 (2.2%)	2 (1.7%)	7 (1.9%)
Intraoperative complications	0 (0%)	0 (0%)	2 (1.7%)	2 (0.5%)
Postoperative complications				
Clavien-Dindo ≥ III	15 (9.7%)	10 (10.8%)	7 (5.8%)	32 (8.7%)
Clavien-Dindo I - II	23 (14.8%)	9 (9.7%)	15 (12.4%)	47 (12.7%)
Anastomotic leakage	6 (3.9%)	5 (5.4%)	2 (1.7%)	13 (3.5%)
30-day reoperation	12 (7.7%)	11 (11.8%)	8 (6.6%)	31 (8.4%)
30-day mortality	3 (1.9%)	1 (1.1%)	1 (0.8%)	5 (1.4%)
Postoperative length of hospital stay				
Mean (SD)	10.4 (6.38)	8.84 (4.88)	8.31 (3.51)	9.34 (5.29)
Median [Q1, Q3]	8.00 [7.00, 12.0]	8.00 [6.00, 10.0]	7.00 [6.00, 9.00]	8.00 [6.00, 11.0]

### Operative time

The overall median operative time was 185 minutes (Q1-Q3: 150–237). Surgeon 1 demonstrated the longest operative times overall, with a median of 205 minutes (Q1-Q3: 169–259), compared with 180 minutes (Q1-Q3: 149–216) for Surgeon 2 and 160 minutes (Q1-Q3: 137–209) for Surgeon 3.

Across all surgeons, RLAR had the highest median operative times, ranging from 217 (Q1-Q3: 172–242) to 242 (Q1-Q3: 191–296) minutes. RRC had the lowest median operative times, including a median of 149 (Q1-Q3: 135–170) minutes for Surgeon 3, whereas RAR showed intermediate operative times (**Table 3**).

**Table 3:** Operative time according to surgeon and procedure.

Variable	Surgeon 1	Surgeon 2	Surgeon 3	Overall
Operative time (min)				
Mean (SD)	220 (70.2)	190 (53.9)	176 (56.6)	198 (64.9)
Median [Q1, Q3]	205 [169, 259]	180 [149, 216]	160 [137, 209]	185 [150, 237]
Robotic right colectomy (RRC)				
Mean (SD)	188 (35.6)	173 (46.0)	157 (36.4)	174 (41.2)
Median [Q1, Q3]	185 [162, 213]	155 [140, 194]	149 [135, 170]	165 [142, 196]
Robotic anterior resection (RAR)				
Mean (SD)	176 (45.6)	179 (37.1)	150 (43.7)	166 (44.2)
Median [Q1, Q3]	165 [141, 206]	173 [157, 208]	150 [119, 166]	161 [137, 191]
Robotic low anterior resection (RLAR)				
Mean (SD)	250 (74.1)	229 (65.1)	215 (57.8)	236 (69.7)
Median [Q1, Q3]	242 [191, 296]	218 [202, 264]	217 [172, 242]	233 [184, 281]

**Figure 1:**
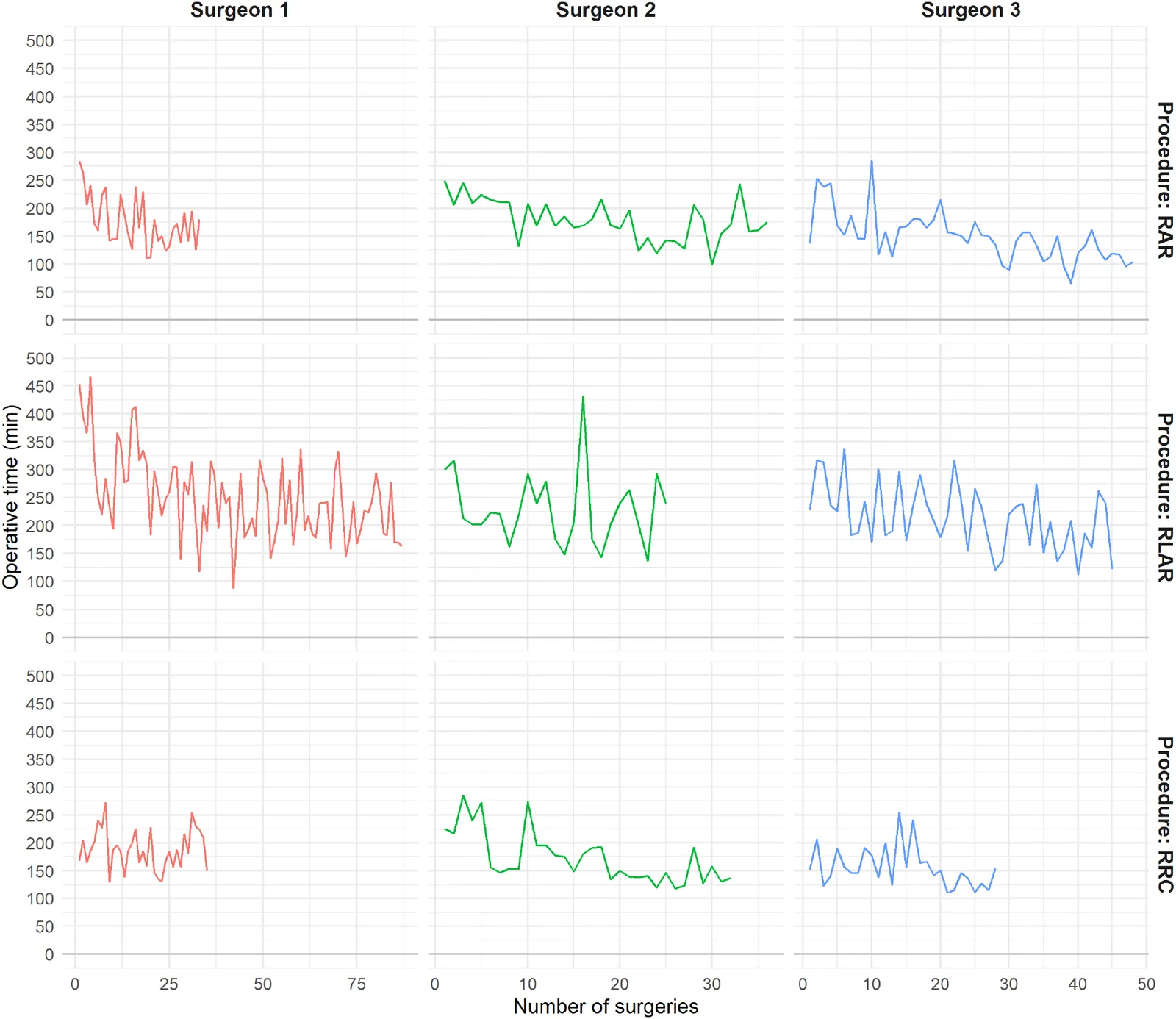
Operative time across consecutive procedures by surgeon and procedure type.

Operative time trends across consecutive procedures are illustrated in **Figure 1** and **Supplementary Figure 1**.

### Histopathological and oncological outcomes

Histopathological analysis included 318 malignant cases. Most tumors were classified as pT3 (47.8%), while nodal-negative disease (pN0) was observed in 62.3% of patients. UICC stage IV disease was present in 16.7% of cases.

R0 resection was achieved in 97.5% of malignant cases, with a median lymph node yield of 23 (Q1-Q3: 17–33). In RLAR procedures, complete or near-complete TME quality was achieved in the majority of patients, and negative circumferential resection margins were observed in 96.8% of cases. In RRC procedures, complete CME quality was reported in 91.6% of cases.

Histopathological and oncological outcomes stratified by surgeon are presented descriptively in **Table 4**.

**Table 4:** Histopathological and oncological outcomes.

Variable	Surgeon 1 (n=149)	Surgeon 2 (n=68)	Surgeon 3 (n=101)	Overall (n=318)
pT Stage				
pT0	7 (4.7%)	2 (2.9%)	2 (2.0%)	11 (3.5%)
pT1	29 (19.5%)	10 (14.7%)	9 (8.9%)	48 (15.1%)
pT2	34 (22.8%)	12 (17.6%)	20 (19.8%)	66 (20.8%)
pT3	66 (44.3%)	27 (39.7%)	59 (58.4%)	152 (47.8%)
pT4	13 (8.7%)	17 (25.0%)	11 (10.9%)	41 (12.9%)
pN Stage				
pN0	97 (65.1%)	42 (61.8%)	59 (58.4%)	198 (62.3%)
pN1	33 (22.1%)	17 (25.0%)	26 (25.7%)	76 (23.9%)
pN2	18 (12.1%)	9 (13.2%)	16 (15.8%)	43 (13.5%)
pN3	1 (0.7%)	0 (0%)	0 (0%)	1 (0.3%)
M Stage				
M0	130 (87.2%)	54 (79.4%)	80 (79.2%)	264 (83.0%)
M1	19 (12.8%)	14 (20.6%)	21 (20.8%)	54 (17.0%)
UICC Stage				
0	7 (4.7%)	2 (2.9%)	1 (1.0%)	10 (3.1%)
I	52 (34.9%)	19 (27.9%)	24 (23.8%)	95 (29.9%)
II	36 (24.2%)	18 (26.5%)	30 (29.7%)	84 (26.4%)
III	36 (24.2%)	15 (22.1%)	25 (24.8%)	76 (23.9%)
IV	18 (12.1%)	14 (20.6%)	21 (20.8%)	53 (16.7%)
Resection status				
R0	145 (97.3%)	66 (97.1%)	99 (98.0%)	310 (97.5%)
R1	4 (2.7%)	2 (2.9%)	2 (2.0%)	8 (2.5%)
Retrieved lymph nodes				
Mean (SD)	26.1 (11.5)	25.4 (10.2)	26.7 (12.4)	26.1 (11.5)
Median [Q1, Q3]	24.0 [17.0, 32.0]	22.0 [17.8, 33.3]	23.0 [18.0, 33.0]	23.0 [17.0, 33.0]
RRC - CME quality				
Grade 0	31/35 (88.6%)	28/32 (87.5%)	28/28 (100%)	87/95 (91.6%)
Grade 1	4/35 (11.4%)	4/32 (12.5%)	0/28 (0%)	8/95 (8.4%)
RLAR - TME quality				
complete	79/87 (90.8%)	24/25 (96.0%)	38/45 (84.4%)	141/157 (89.8%)
nearly complete	4/87 (4.6%)	1/25 (4.0%)	4/45 (8.9%)	9/157 (5.7%)
incomplete	4/87 (4.6%)	0/25 (0%)	3/45 (6.7%)	7/157 (4.5%)
RLAR - CRM status				
negative	84/87 (96.6%)	24/25 (96.0%)	44/45 (97.8%)	152/157 (96.8%)
positive	3/87 (3.4%)	1/25 (4.0%)	1/45 (2.2%)	5/157 (3.2%)

### Learning curve analysis

**Figure 2:**
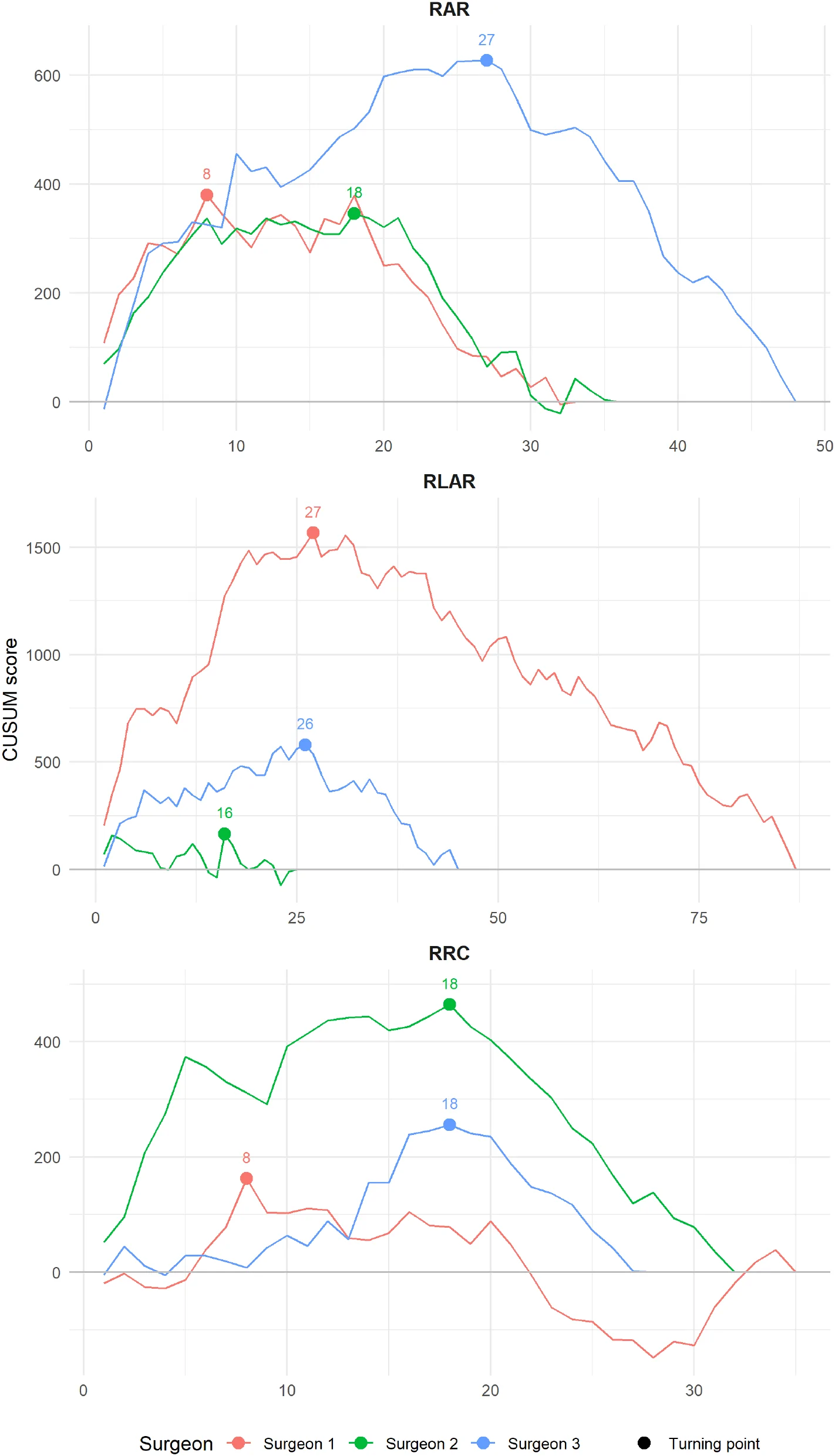
Surgeon- and procedure-specific CUSUM curves of operative time. CUSUM curves of consecutive skin-to-skin operative times for RAR, RLAR, and RRC are shown for each surgeon. Turning points indicate changes in the cumulative operative-time trend.

CUSUM analysis demonstrated substantial surgeon- and procedure-specific variability in operative-time trajectories during institutional implementation of robotic colorectal surgery (**Figure 2**). RRC generally showed earlier CUSUM turning points, whereas RLAR was characterized by greater variability in operative-time trajectories across surgeons. However, the timing of CUSUM turning points differed between individual surgeons and procedures.

### Robotic right colectomy

RRC showed CUSUM turning points after 8 cases for Surgeon 1 and approximately 18 cases for Surgeons 2 and 3. Following these turning points, the CUSUM curves showed an overall downward trend, indicating a shift toward shorter operative times relative to the surgeon-specific reference values.

### Robotic anterior resection

RAR showed differences in CUSUM trajectories between surgeons. Turning points were identified after approximately 8 cases for Surgeon 1, 18 cases for Surgeon 2, and 27 cases for Surgeon 3. Following the respective turning points, the CUSUM curves showed an overall downward trend.

### Robotic low anterior resection

RLAR showed the highest median operative times and marked inter-surgeon variability in CUSUM trajectories. Turning points were identified after approximately 27 cases for Surgeon 1, 16 cases for Surgeon 2, and 26 cases for Surgeon 3. Thus, unlike Surgeons 1 and 3, Surgeon 2 showed an earlier RLAR turning point than for RAR and RRC.

### Overall comparison of CUSUM trajectories

CUSUM trajectories and visually identified turning points varied between surgeons and procedures. Turning points ranged from 8 to 27 procedures, with substantial inter-surgeon variability across procedure types. RRC turning points ranged from 8 to 18 cases, RAR turning points from 8 to 27 cases, and RLAR turning points from 16 to 27 cases. Notably, Surgeon 2 showed an earlier turning point for RLAR than for RAR and RRC, illustrating that higher operative times and greater CUSUM variability did not necessarily correspond to a later turning point. Detailed CUSUM turning points according to surgeon and procedure are summarized in **Table 5**.

**Table 5:** CUSUM turning points according to surgeon and procedure.

Surgeon	Procedure	n	Turning point (case no.)
Surgeon 1	RRC	35	8
	RAR	33	8
	RLAR	87	27
Surgeon 2	RRC	32	18
	RAR	36	18
	RLAR	25	16
Surgeon 3	RRC	28	18
	RAR	48	27
	RLAR	45	26

### Risk-adjusted CUSUM analysis

**Figure 3:**
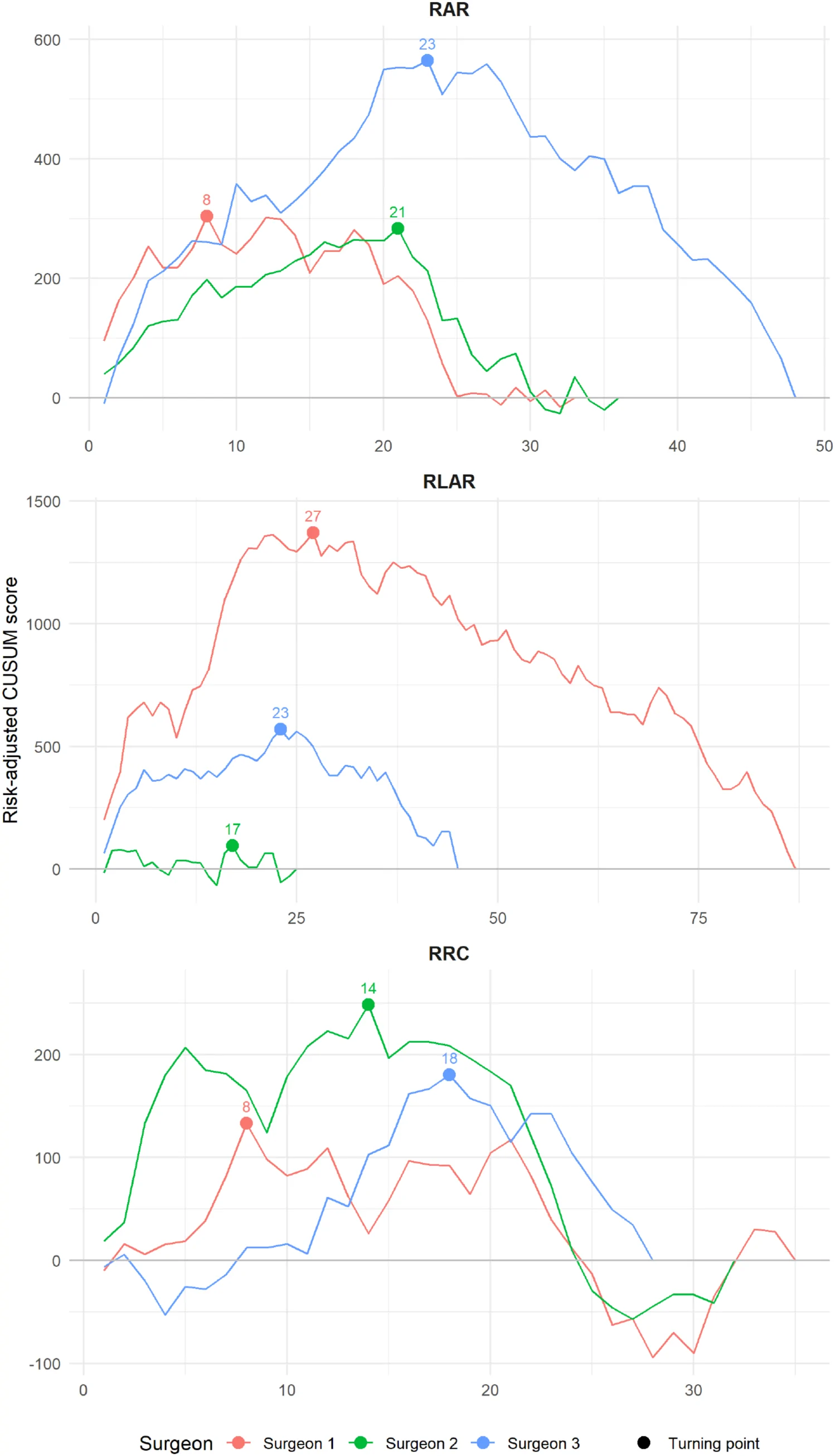
Surgeon- and procedure-specific risk-adjusted CUSUM curves of operative time. RA-CUSUM curves show cumulative deviations between observed and expected operative times after adjustment for relevant covariates. Turning points indicate changes in the cumulative risk-adjusted operative-time trend.

RA-CUSUM analysis demonstrated surgeon- and procedure-specific variation in adjusted operative-time trajectories after accounting for the covariates included in the regression model (**Figure 3**). The overall patterns were broadly consistent with the unadjusted CUSUM analysis, although differences in curve shape and cumulative deviation were observed after risk adjustment. RA-CUSUM findings were interpreted as changes in adjusted operative-time trends and not as measures of procedural safety or surgical proficiency.

## Discussion

The present study builds on our previous work on the institutional implementation of robotic colorectal surgery. In our initial single-surgeon series, we identified procedure-specific differences in operative learning trajectories within a structured dual-console training environment (6, 7). The present multi-surgeon analysis extends these observations by examining whether similar operative-time patterns are observed across different surgeons performing multiple robotic colorectal procedures within the same institutional program.

The principal finding is that operative-time CUSUM trajectories varied substantially between surgeons and procedures despite a broadly standardized institutional training and operative framework. Differences were observed in curve shape, cumulative deviation, and visually identified turning points. These findings indicate that surgeon- and procedure-specific CUSUM patterns should not be interpreted as universally applicable numerical thresholds, but rather as descriptive measures of operative-time adaptation that require procedure- and surgeon-specific interpretation (12).

### Procedure-specific learning curves

A major strength of the present study is the procedure-specific assessment of operative-time learning trajectories rather than pooling different colorectal procedures. This distinction is relevant because robotic colorectal surgery encompasses procedures with substantially different operative characteristics and technical requirements.

### Robotic right colectomy

RRC showed relatively early CUSUM turning points, ranging from 8 to 18 procedures, although the timing differed between surgeons. The chronological structure of our institutional robotic program may have contributed to these patterns. Robotic implementation initially focused predominantly on rectal surgery, and Surgeons 1 and 3 had already gained experience with robotic rectal procedures before introducing RRC. In contrast, Surgeon 2 introduced RRC, RAR, and RLAR within a narrower time frame. Prior exposure to the robotic platform may therefore have influenced subsequent operative-time adaptation during RRC, although such a transfer effect cannot be directly assessed from the present data.

Previous studies have similarly reported improvements in operative efficiency during the early adoption of RRC (13–15). Our findings therefore suggest that the sequence in which different robotic procedures are introduced may be relevant when interpreting surgeon-specific operative-time trajectories.

### Robotic anterior resection

RAR showed intermediate operative-time patterns, with visually identified CUSUM turning points ranging from approximately 8 to 27 procedures. Compared with RLAR, RAR generally involves dissection in the upper rectum with PME rather than deep pelvic TME, which may contribute to differences in operative-time trajectories. However, the observed inter-surgeon variation may also reflect differences in patient characteristics, case complexity, and prior robotic experience. Previous studies have similarly reported progressive changes in operative efficiency during the implementation of robotic colorectal surgery (2, 16).

### Robotic low anterior resection

RLAR was associated with the highest median operative times and marked inter-surgeon variability in CUSUM trajectories. Visually identified turning points ranged from approximately 16 to 27 procedures. Importantly, however, a higher operative time or greater cumulative CUSUM deviation did not necessarily correspond to a later turning point. Surgeon 2, for example, showed an earlier turning point for RLAR than for RAR and RRC. This illustrates that operative duration, CUSUM trajectory, and timing of the turning point represent distinct aspects of operative-time adaptation and should not be interpreted interchangeably.

The variability observed during RLAR may partly relate to the technical characteristics of low rectal surgery, including deep pelvic TME, restricted pelvic anatomy, and variation in patient and tumor characteristics. Previous studies have also reported heterogeneous operative learning trajectories during robotic rectal surgery (17–20). However, the present data do not establish that RLAR requires a universally longer learning period or a specific number of procedures to achieve proficiency.

### Perioperative and oncological outcomes during implementation

Overall perioperative and oncological outcomes were acceptable across the study cohort despite heterogeneous operative-time trajectories. Conversion, major postoperative complication, and anastomotic leakage rates were low, while oncological outcomes were characterized by high R0 resection rates, lymph node yield, TME/CME specimen quality, and CRM negativity. Similar overall outcomes during the implementation of robotic colorectal surgery have been reported previously (3, 13, 18). However, as clinical and oncological outcomes were not analyzed longitudinally according to learning phase or CUSUM turning points, the present study cannot determine whether these outcomes remained stable throughout the learning process.

### Structured robotic training and dual-console implementation

Robotic colorectal surgery was implemented within a structured institutional training program comprising simulation training, bedside assistance, formal proctoring, and progressive operative participation before transition to independent robotic surgery. Similar structured approaches to the implementation and learning process of robotic rectal surgery have been described in previous single- and multicenter studies (17, 18, 20).

During the initial implementation phase, selected procedures were performed using the dual-console platform, allowing a second surgeon to observe the procedure in real time and occasionally perform selected operative steps under supervision. This setting provided opportunities for supervised participation and progressive familiarization with robotic colorectal procedures (21).

However, the present study was not designed to evaluate the independent effect of the structured training pathway or dual-console use, and no causal relationship between these measures and clinical outcomes can be established. The training pathway and dual-console strategy should therefore be regarded as components of the institutional implementation framework rather than factors proven to account for the observed outcomes.

### Limitations

Several limitations should be acknowledged. First, the retrospective single-center design may limit generalizability to institutions with different case volumes, robotic experience, or training structures. Second, procedural distribution and oncological case mix differed between surgeons, which may have influenced surgeon- and procedure-specific operative-time trajectories and limits direct comparisons between surgeons. Third, operative time represents only one dimension of surgical performance and does not capture other aspects of surgical proficiency or clinical outcomes. Moreover, CUSUM turning points were identified by visual inspection and should be regarded as descriptive changes in operative-time trends rather than validated thresholds of surgical proficiency. Fourth, surgeons initiating robotic surgery later during institutional implementation may have benefited from increasing institutional and operative team experience. In addition, the da Vinci Xi and da Vinci 5 systems were introduced toward the end of the study period; however, case-level platform use was not systematically recorded, precluding separate assessment of potential platform-related effects on operative time. Fifth, the inclusion of patients with diverticular disease in the RAR cohort may have introduced additional procedural heterogeneity despite a standardized operative approach. Sixth, perioperative and oncological outcomes were assessed descriptively and were not analyzed longitudinally according to learning phase or CUSUM turning points; therefore, their stability throughout the learning process cannot be determined from the present study. A further limitation is the relatively small number of cases in some surgeon–procedure groups in relation to the number of covariates included in the risk-adjustment models. Although basic regression diagnostics did not indicate major violations of the underlying model assumptions, the expected operative times derived from models with small sample sizes may be associated with greater uncertainty and potential overfitting. Therefore, the risk-adjusted operative times and corresponding CUSUM curves should be interpreted with caution, particularly for surgeon–procedure combinations with fewer cases. Finally, long-term oncological and functional outcomes were not evaluated.

## Conclusion

Operative-time learning trajectories in robotic colorectal surgery demonstrated substantial variability according to both surgeon and procedure, even within a standardized institutional robotic program. RRC generally showed earlier CUSUM turning points, whereas RLAR was characterized by higher operative times and greater inter-surgeon variability in CUSUM trajectories. Overall perioperative and oncological outcomes were acceptable across the study cohort. These findings suggest that operative-time adaptation differs between surgeons and procedures and should not be interpreted using universally applicable case-number thresholds alone.

## Data Availability

The original contributions presented in the study are included in the article/supplementary material. Further inquiries can be directed to the corresponding author.
