# Supplementary material for: Surgeon- and procedure-specific variability in operative-time learning curves during parallel implementation of robotic colorectal surgery
Source: Front Oncol. 2026 Sep 11;16:1905964. doi: 10.3389/fonc.2026.1905964 (PMC13612354; doi:10.3389/fonc.2026.1905964)
Supplement: Supplementary file 1 [file DataSheet1.pdf]

Supplementary Material

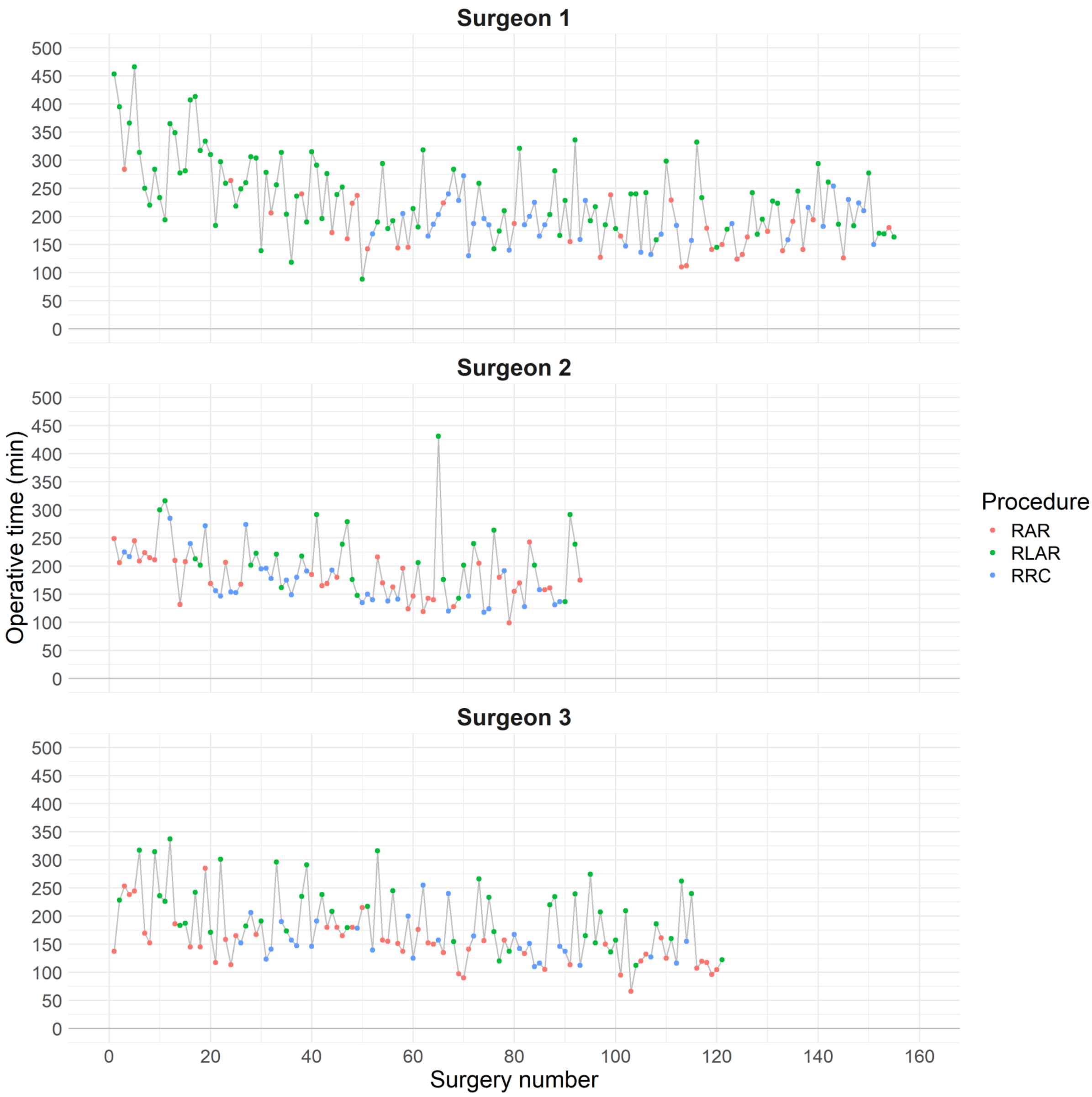

Figure S1: ***Chronological distribution of operative times by surgeon and procedure.*** *Individual skin-to-skin operative times are shown in chronological order for Surgeon 1 (n=155), Surgeon 2 (n=93), and Surgeon 3 (n=121). Data points indicate the procedure performed: robotic anterior resection (RAR), robotic low anterior resection (RLAR), and robotic right colectomy (RRC). The figure illustrates the raw operative-time distribution and procedural case mix across each surgeon's consecutive case sequence.*

Table S1: Descriptive statistics of predictors included in the multivariable linear regression models, by surgeon and procedure

	Surgeon 1			Surgeon 2			Surgeon 3		
	RAR (N=33)	RLAR (N=87)	RRC (N=35)	RAR (N=36)	RLAR (N=25)	RRC (N=32)	RAR (N=48)	RLAR (N=45)	RRC (N=28)
**Age (in years)**									
Mean (SD)	70.5 (10.9)	69.0 (11.8)	72.1 (11.2)	65.1 (14.6)	70.7 (11.7)	74.8 (9.21)	67.3 (12.7)	67.8 (11.9)	72.9 (8.13)
Median [Q1, Q3]	70.0 [64.0, 80.0]	71.0 [60.5, 79.0]	74.0 [66.5, 80.5]	62.0 [57.0, 75.5]	70.0 [65.0, 79.0]	78.0 [68.8, 83.0]	68.5 [58.8, 76.3]	68.0 [61.0, 76.0]	74.5 [67.8, 80.0]
**Sex**									
**Female**	12 (36.4%)	30 (34.5%)	20 (57.1%)	21 (58.3%)	11 (44.0%)	14 (43.8%)	21 (43.8%)	12 (26.7%)	14 (50.0%)
Male	21 (63.6%)	57 (65.5%)	15 (42.9%)	15 (41.7%)	14 (56.0%)	18 (56.3%)	27 (56.3%)	33 (73.3%)	14 (50.0%)
**BMI**									
Mean (SD)	27.6 (4.42)	26.2 (4.40)	27.5 (4.90)	29.9 (6.53)	26.6 (6.08)	25.6 (3.68)	26.4 (4.15)	26.2 (5.23)	26.2 (4.80)
Median [Q1, Q3]	28.0 [24.0, 30.0]	25.0 [23.0, 28.0]	27.0 [24.0, 30.0]	29.0 [25.8, 32.3]	26.0 [23.0, 28.0]	25.5 [24.0, 28.0]	26.0 [24.0, 28.0]	26.0 [23.0, 28.0]	25.5 [22.8, 29.0]
**ASA Score**									
I	2 (6.1%)	1 (1.1%)	2 (5.7%)	3 (8.3%)	0 (0%)	0 (0%)	7 (14.6%)	1 (2.2%)	1 (3.6%)
II	13 (39.4%)	39 (44.8%)	18 (51.4%)	17 (47.2%)	8 (32.0%)	11 (34.4%)	23 (47.9%)	17 (37.8%)	11 (39.3%)
III	18 (54.5%)	43 (49.4%)	13 (37.1%)	15 (41.7%)	14 (56.0%)	20 (62.5%)	17 (35.4%)	23 (51.1%)	15 (53.6%)
IV	0 (0%)	4 (4.6%)	2 (5.7%)	1 (2.8%)	3 (12.0%)	1 (3.1%)	1 (2.1%)	4 (8.9%)	1 (3.6%)
**Previous abdominal surgery**	9 (27.3%)	23 (26.4%)	10 (28.6%)	14 (38.9%)	6 (24.0%)	11 (34.4%)	13 (27.1%)	9 (20.0%)	4 (14.3%)
**Smoking**	6 (18.2%)	14 (16.1%)	5 (14.3%)	8 (22.2%)	6 (24.0%)	7 (21.9%)	10 (20.8%)	9 (20.0%)	8 (28.6%)

Model diagnostics

Surgeon 1

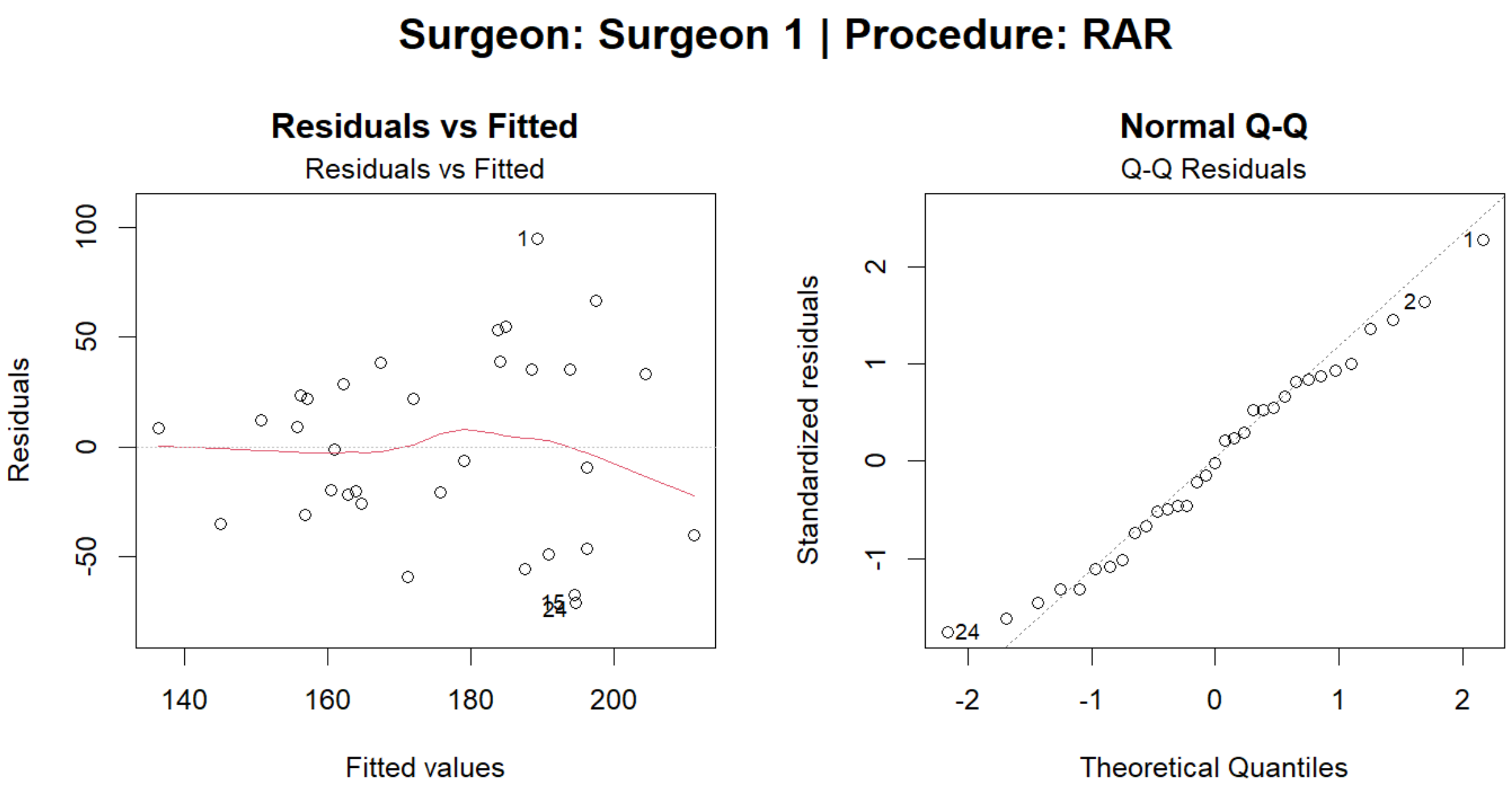

Figure S2: Diagnostic plots for the multivariable linear regression model of operative time for robotic anterior resection (RAR) performed by Surgeon 1.

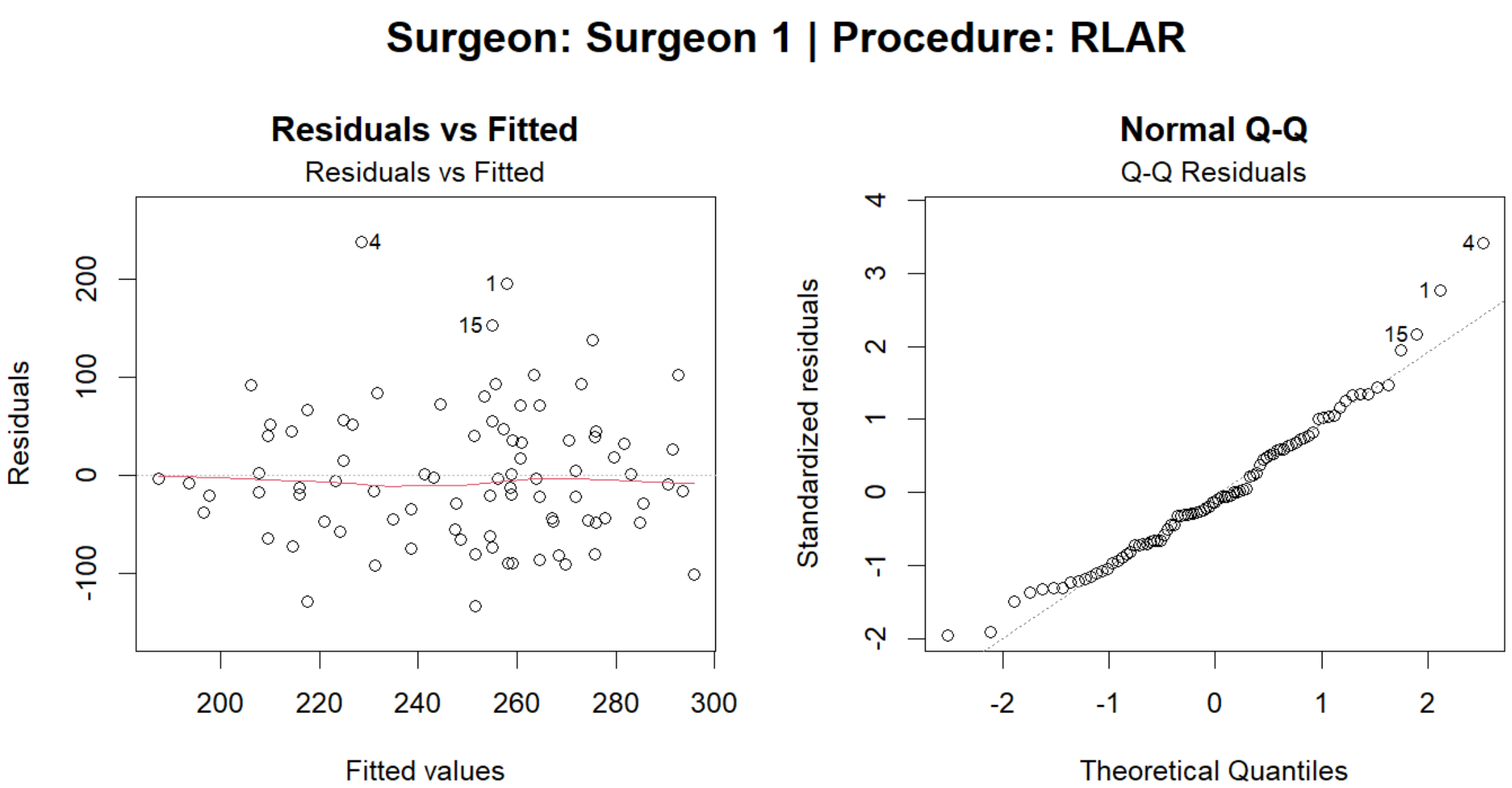

Figure S3: Diagnostic plots for the multivariable linear regression model of operative time for robotic low anterior resection (RLAR) performed by Surgeon 1.

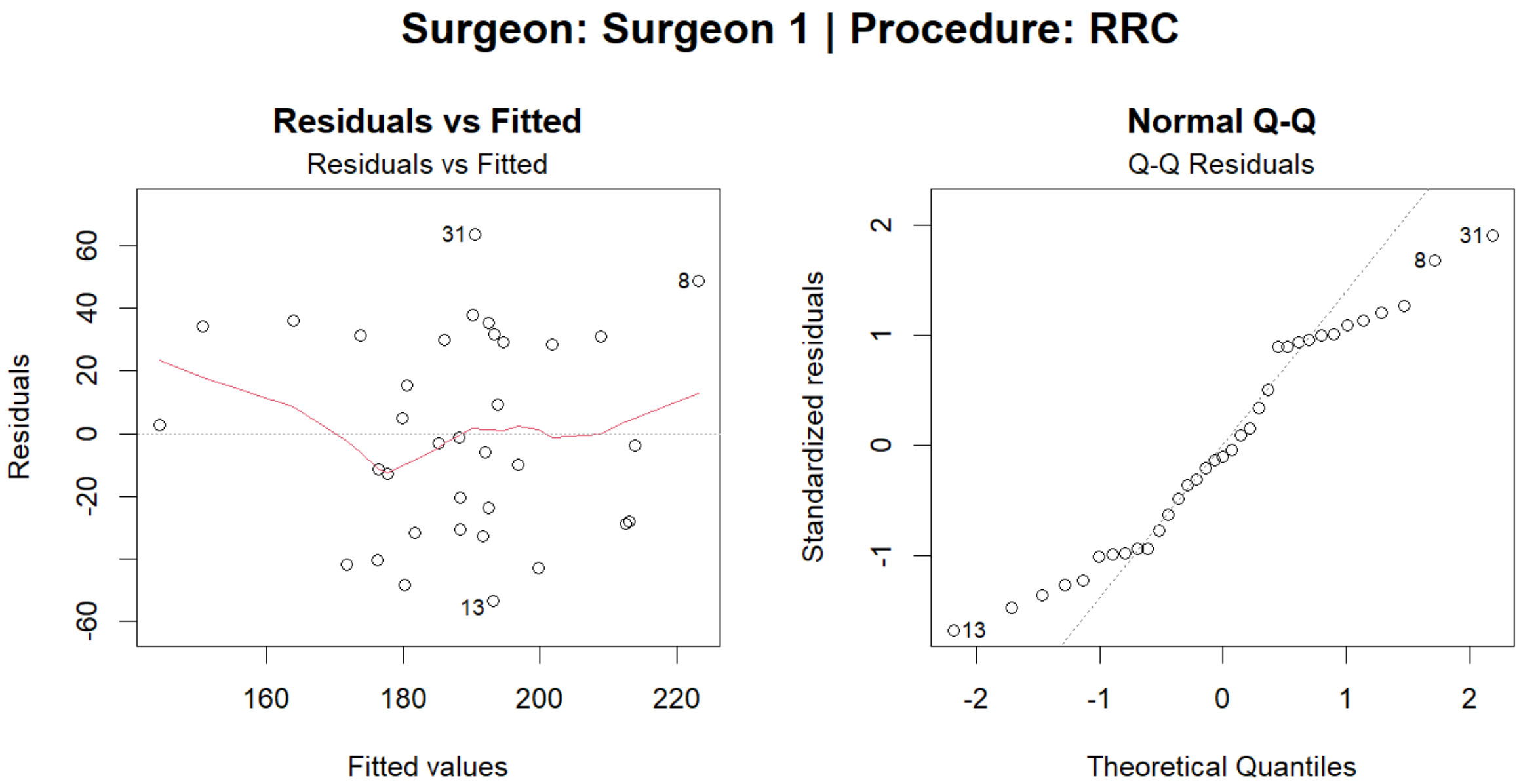

Figure S4: Diagnostic plots for the multivariable linear regression model of operative time for robotic right colectomy (RRC) performed by Surgeon 1

Surgeon 2

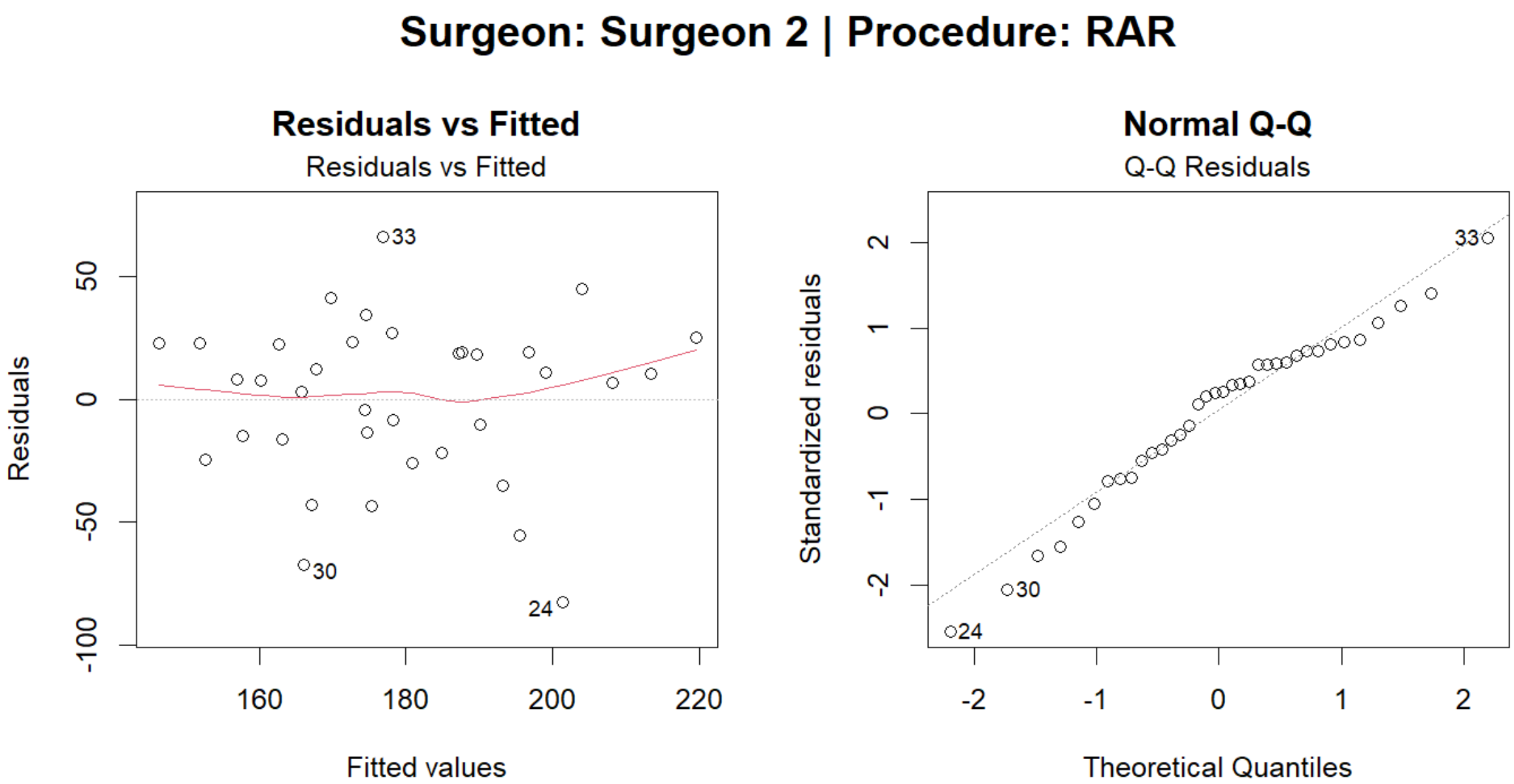

Figure S5: Diagnostic plots for the multivariable linear regression model of operative time for robotic anterior resection (RAR) performed by Surgeon 2.

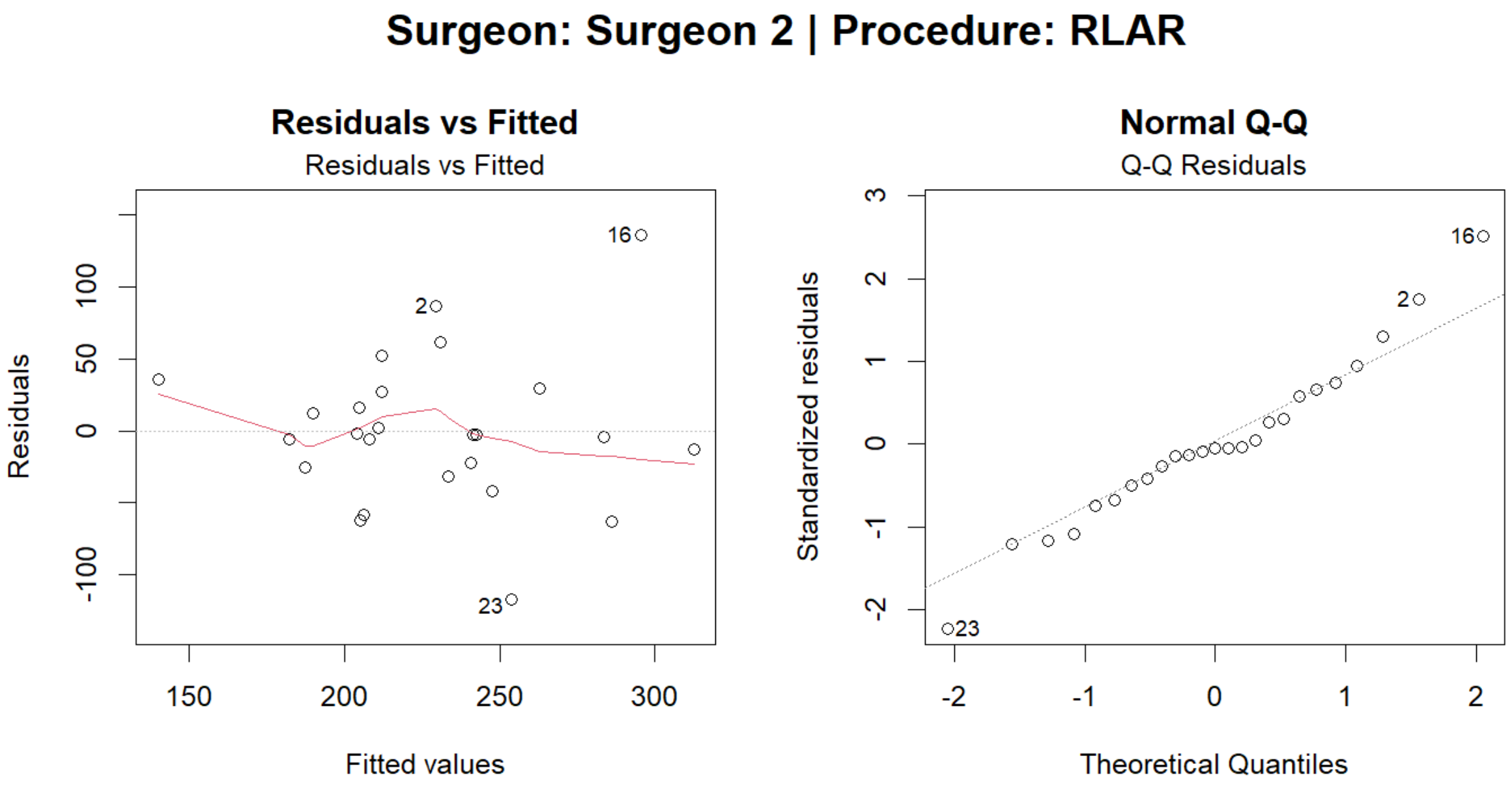

Figure S6: Diagnostic plots for the multivariable linear regression model of operative time for robotic low anterior resection (RLAR) performed by Surgeon 2.

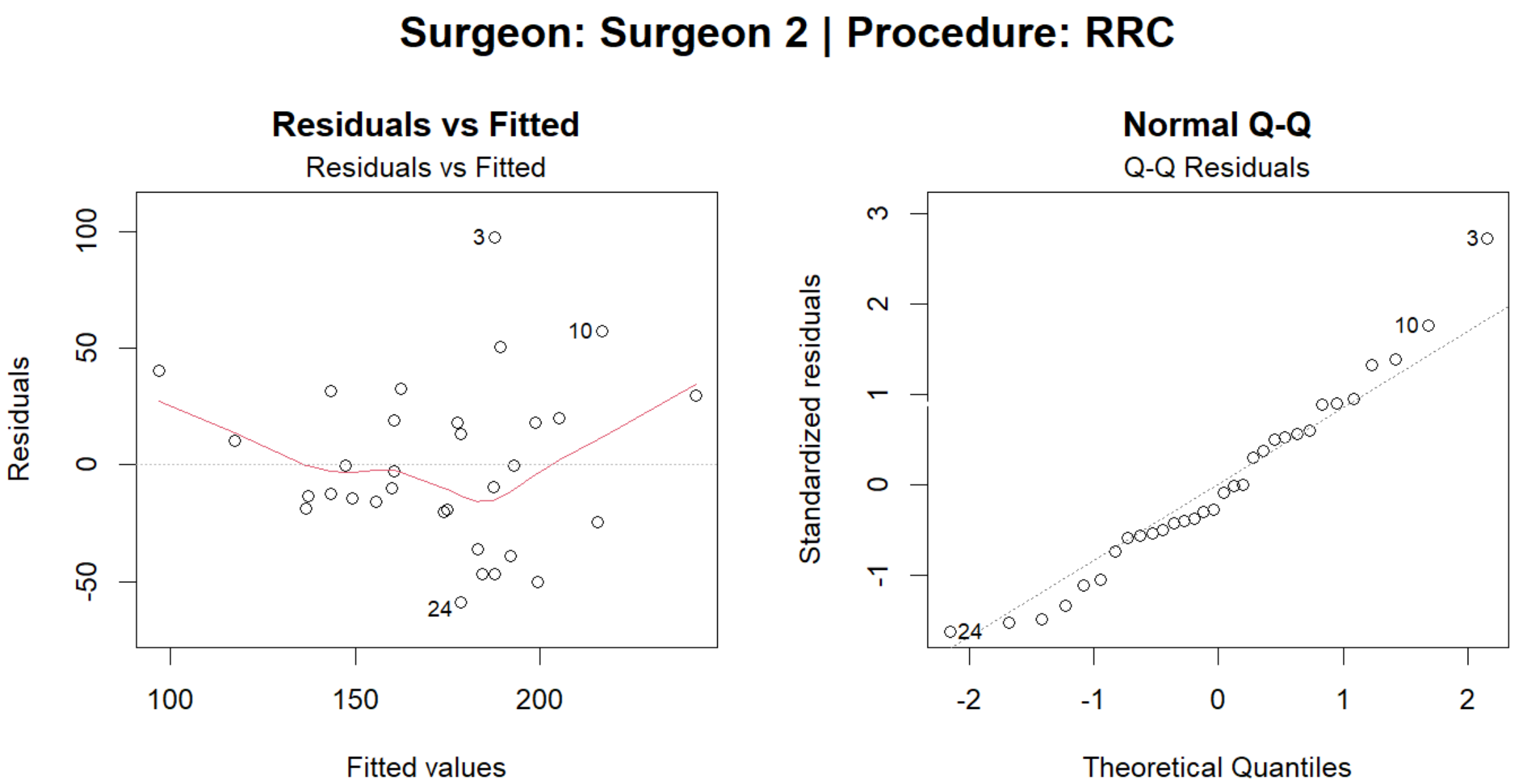

Figure S7: Diagnostic plots for the multivariable linear regression model of operative time for robotic right colectomy (RRC) performed by Surgeon 2.

Surgeon 3

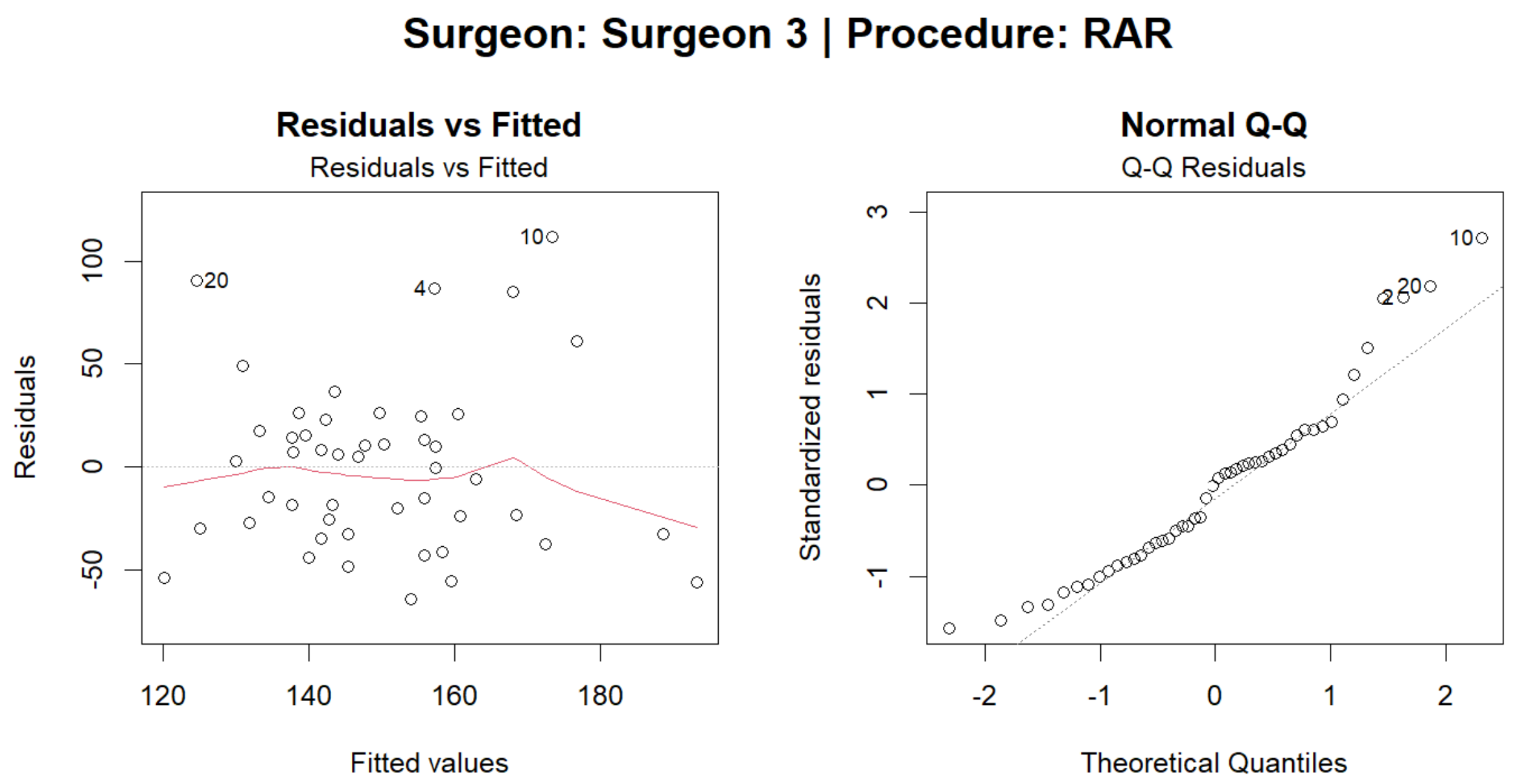

Figure S8: Diagnostic plots for the multivariable linear regression model of operative time for robotic anterior resection (RAR) performed by Surgeon 3.

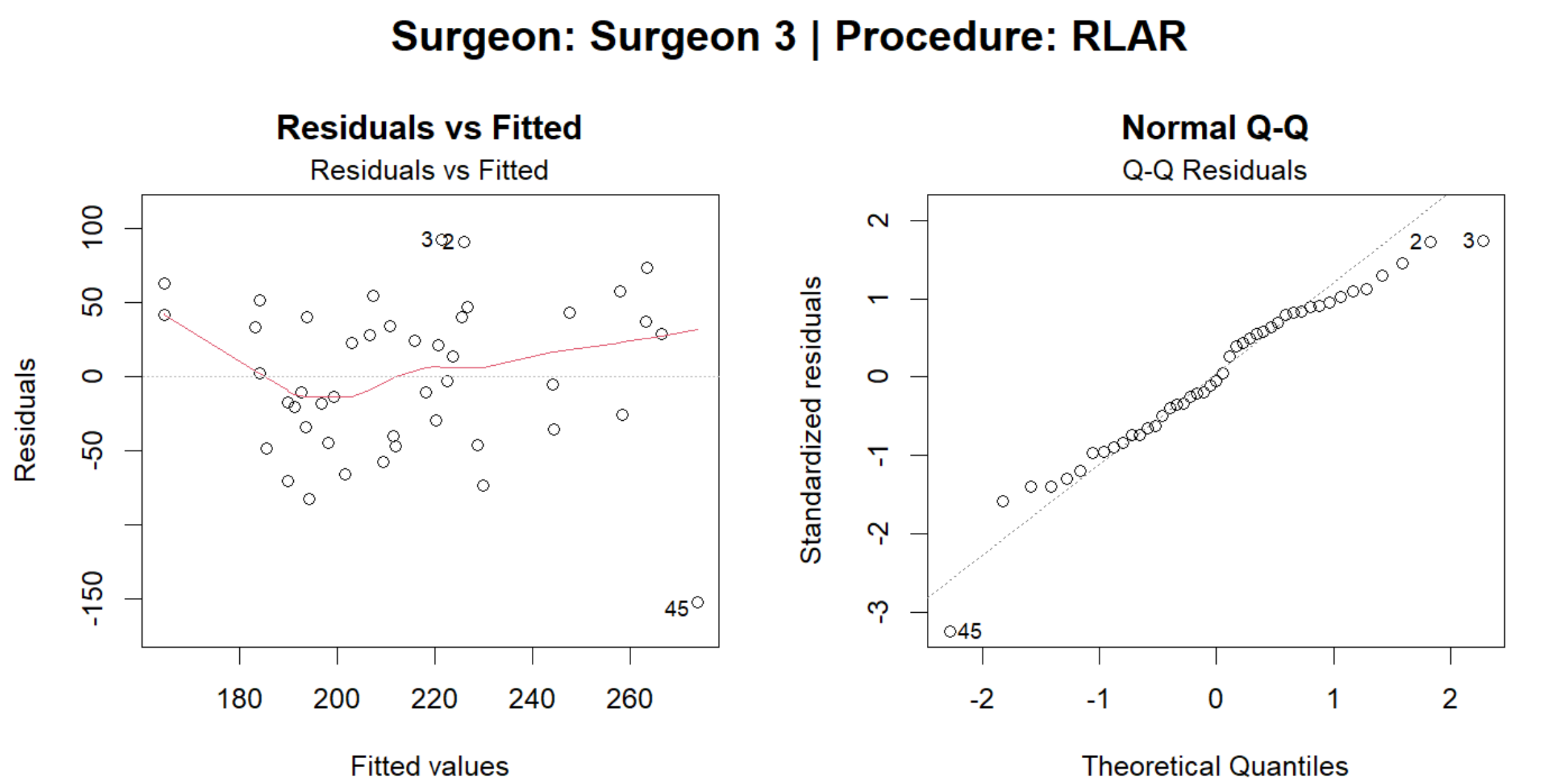

Figure S9: Diagnostic plots for the multivariable linear regression model of operative time for robotic low anterior resection (RLAR) performed by Surgeon 3.

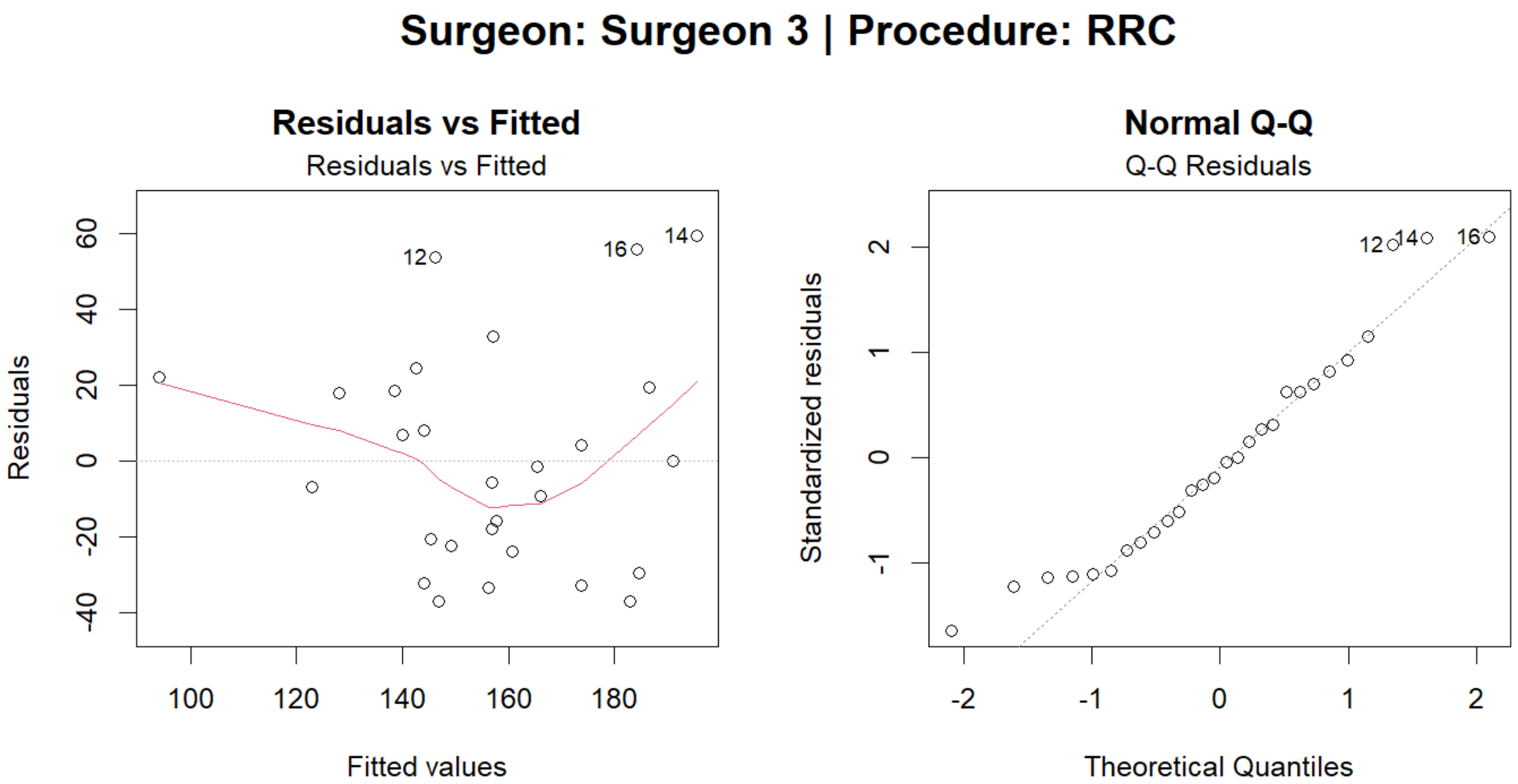

Figure S10: Diagnostic plots for the multivariable linear regression model of operative time for robotic right colectomy (RRC) performed by Surgeon 3.
